# Incompletely Reported Important Methodological Details and Inaccurate Description of the Formulation That the Control Arms Received in a Gardasil Vaccine Trial

**DOI:** 10.1128/mSphere.00770-20

**Published:** 2020-11-04

**Authors:** Florence Bourgeois, Peter Doshi, Kyungwan Hong, Tom Jefferson, Mark Jones, Haeyoung Lee, Anisa Rowhani-Farid, Larissa Shamseer, O’Mareen Spence

**Affiliations:** a Department of Pediatrics, Harvard Medical School, Boston, Massachusetts, USA; b Department of Pharmaceutical Health Services Research, University of Maryland School of Pharmacy, Baltimore, Maryland, USA; c Centre for Evidence-Based Medicine, University of Oxford, Oxford, United Kingdom; d Nordic Cochrane Centre, Copenhagen, Denmark; e Institute for Evidence-based Healthcare, Bond University, Gold Coast, Queensland, Australia; f University of Ottawa, Ottawa, Ontario, Canada; University of Michigan-Ann Arbor

**Keywords:** drug regulation, drug safety, evidence-based medicine, transparency

## LETTER

The Restoring Invisible and Abandoned Trials (RIAT) initiative is an international effort concerned with two fundamental problems in the scientific literature on clinical trials: many trials are inaccurately or incompletely reported in journal publications and not all trials conducted are even published. RIAT aims to address these problems by offering a methodology that allows other people to responsibly correct the record (1).

We are writing with concerns about inaccurate and incomplete reporting in a trial published in *Clinical and Vaccine Immunology*, as well as ethical concerns about the informed consent process.

In their study of quadrivalent human papillomavirus vaccine (NCT00092482) (2), the authors stated that they conducted a “placebo-controlled” trial. However, control arm participants did not receive a “placebo” or an “inactive solution,” the descriptions provided in the informed consent forms for this trial. Instead, they received an injection containing amorphous aluminum hydroxyphosphate sulfate (AAHS), a proprietary adjuvant added to enhance immune response (3). The use of the term “placebo” to describe an active and reactogenic comparator like AAHS inaccurately describes the formulation that the control arm received and may also have obscured an accurate assessment of vaccine safety. Further, the publication does not report the rationale for the selection of AAHS control, an important omission that constitutes underreporting of important methodological details.

According to the manufacturer’s clinical study report for this trial, the rationale for selecting AAHS adjuvant as the control was as follows:

“Aluminum adjuvant was chosen as the appropriate control for the qHPV vaccine for the following reasons:

The inclusion of aluminum adjuvant in both vaccine and placebo preserved the blinding of the study because it allowed the vaccine and placebo to be visually indistinguishable; and

The safety profile of Merck’s aluminum adjuvant is well characterised. On the other hand, the safety profile of the HPV 6, 11, 16 and 18 L1 VLPs required further evaluation in humans. By using placebo that contained a dose of aluminum adjuvant that was identical to the dose included in the qHPV vaccine, it was possible to assess the safety profile attributable to the HPV 6, 11, 16 and 18 L1 VLP component of the vaccine” (3).

Thus, the manufacturer’s stated rationale for selecting AAHS as a control (to characterize the safety of human papillomavirus [HPV]-like particles) lacks clinical relevance, and a nonplacebo control may have obscured an accurate assessment of quadrivalent HPV vaccine safety (3). We have documented all of these issues elsewhere (3).
